# Advanced Footwear Technology in Non-Elite Runners: A Survey of Training Practices and Reported Outcomes

**DOI:** 10.3390/sports12120356

**Published:** 2024-12-20

**Authors:** Matteo Bonato, Federica Marmondi, Emanuela Luisa Faelli, Chiara Pedrinelli, Luigi Ferraris, Luca Filipas

**Affiliations:** 1 Department of Biomedical Sciences for Health, Università degli Studi di Milano, Via Giuseppe Colombo 71, 20133 Milan, Italy; chiara.pedrinelli@studenti.unimi.it (C.P.); luca.filipas@unimi.it (L.F.); 2 Laboratory of Movement and Sport Sciences (LaMSS), IRCCS Istituto Ortopedico Galeazzi, Via Cristina da Belgioioso 173, 20157 Milan, Italy; 3 Department of Neuroscience, Rehabilitation, Ophthalmology, Genetics and Maternal Child Health, Università degli Studi di Genova, 16132 Genoa, Italy; federica.marmondi@edu.unige.it; 4 Centro Polifunzionale di Scienze Motorie, University of Genoa, 16132 Genoa, Italy; emanuela.faelli@unige.it; 5 Section of Human Physiology, Department of Experimental Medicine, Università degli Studi di Genova, 16132 Genoa, Italy; 6 Ready To Run, Via Carducci, 4, 24020 Torre Boldone, Italy; info@readytorun.it; 7 Department of Endocrinology, Nutrition and Metabolic Diseases, IRCCS MultiMedica, 20157 Milan, Italy

**Keywords:** running, training, footwear technology, musculoskeletal, performance

## Abstract

**Background:** Advanced footwear technology (AFT) has gained popularity among non-elite runners due to its potential benefits in training and competition. This study investigated the training practices and reported outcomes in non-elite runners using AFT. **Methods:** A cross-sectional observational study was conducted with 61 non-elite runners competing in distances ranging from 5 km to marathons. The survey collected data on demographics, training parameters, footwear usage, perceived changes in running mechanics, and self-reported injuries. **Results:** The results revealed a significant positive correlation (R = 0.6, *p* < 0.0001) between years of AFT use and weekly training volume, indicating that more experienced runners are likely to incorporate AFT consistently into their routines. Conversely, a significant negative correlation (R = −0.5, *p* < 0.0001) was found between training volume and the number of weekly sessions using AFT, suggesting a selective approach to footwear use. Participants reported biomechanical changes, such as increased forefoot support (49%) and higher calf muscle activation (44%), alongside a 16% self-reported injury rate, predominantly affecting the calves. **Conclusions:** These findings highlight the importance of proper guidance and gradual adaptation to maximize the benefits of AFT while minimizing injury risks. Future research should explore the long-term impact of AFT on performance and injury prevention through longitudinal studies.

## 1. Introduction

Advanced footwear technology (AFT) shoes were first introduced by Nike in late 2016–early 2017, and they consisted of performance-enhancing shoes that combine lightweight, resilient midsole foams with rigid moderators and pronounced rocker profiles in the soles [1]. Unlike traditional racing flats, AFT shoes are designed to improve running economy and performance through innovations such as enhanced bending stiffness, high-energy return foam, and increased stack height [2]. A recent systematic review stated that most of the recent research focused on investigating the impact of running shoe midsoles, bending stiffness, and heel-to-toe drop on athletic performance [3]. Thanks to these features, elite athletes significantly improved their performances by 4–5% [4], and the progression of world records by males and females starting from 5 km to marathon length was broken [5]. Supporting this, Willwacher et al. [6] analyzed the 100 best yearly performances globally between 2010 and 2022, finding that AFT shoes contributed to systematic improvements in running economy and performance across multiple events. Their findings highlight pronounced benefits in long-distance events, particularly among women, who showed performance gains of 2.2% to 3.5%, compared to 0.7% to 1.4% in men. These characteristics also enabled great achievements such as Eliud Kipchoge’s sub-2-h marathon, which would have been deemed unlikely without technological enhancements regarding AFT [7]. These improvements have been consistently supported by laboratory-based studies, which highlight that AFT reduces the energy cost of running by 2.7% to 4.4%, translating into enhanced performance for elite runners [2,8]. While AFT is now a standard choice for elite athletes during both training and competition, its adoption among non-elite runners is less understood. Bermon [9] observed similar performance in sub-elite and recreational runners, suggesting the broader accessibility of AFT benefits.

The use of AFT shoes during training and competition has introduced novel biomechanical demands of the foot and lower extremities [10]. The biomechanical differences between AFT shoes and standard running footwear were investigated by Hoogkamer et al. [11], who found that runners using AFT shoes had a decrease in cadence and correspondingly longer steps, as well as a longer flight time. The use of AFT has also been associated with higher peak vertical ground reaction forces, vertical impulse per step, differences in ankle and metatarsophalangeal joint mechanics, and reduced peak ankle dorsiflexion during stance, as well as increased peak ankle moments, all of which are biomechanical changes believed to enhance running economy and overall performance, particularly in elite runners [11]. However, the same changes impose additional demands on the foot and lower extremities, which may lead to injury risk if runners fail to adapt properly. According to Tenforde et al. [10], the changes in foot and ankle mechanics introduced via AFT shoe footwear may contribute to the risk of injury. Biomechanical analyses suggest that these improvements come with shifts in running dynamics, such as increased forefoot loading and changes in stride mechanics, which could contribute to overuse injuries, particularly in runners who are not accustomed to these demands [12,13]. Recent studies have begun to explore the impact of footwear technology on injury patterns in non-elite populations, shedding light on its potential benefits and risks. For instance, Theisen et al. [14] highlighted that much of the presumed benefits of advanced footwear technology are based on biomechanical assumptions, rather than robust epidemiological evidence. Similarly, Malisoux et al. [15] emphasized the complexity of the relationship between footwear, training loads, and injury risk, noting that perceived comfort and mechanical properties may not always align with strategies for injury prevention. Furthermore, systematic reviews, such as the review of Sun et al. [16], have shown that specific shoe features, such as midsole stiffness or heel–toe drop, may influence biomechanical variables but currently lack consistent evidence for reducing injury incidence. However, poor research and epidemiology articles on health concerns around using AFT shoes are available. Expressing their current opinion, Hoogkamer et al. [11] illustrated a series of navicular bone stress injuries in two cohorts, including a population of junior track and field athletes and two master athletes competing in endurance events.

Despite the growing popularity of AFT, most existing research focuses on elite athletes, leaving a significant gap in understanding its implications for non-elite runners. The number of non-elite recreational runners steadily increases and represents a substantial portion of the global running community. These athletes are often driven by personal health and wellness goals, rather than competitive ambitions, like running closely to pursue a disciplined and virtuous lifestyle [17]. Non-elite runners differ from elite athletes in several biomechanical characteristics, including reduced cadence, increased ground contact time, and greater variability in stride mechanics. These factors, combined with differences in training practices and access to professional guidance, may contribute to their unique injury risks. Furthermore, non-elite runners face a significant prevalence of running-related injuries, which range from 37 to 79%, depending on the follow-up duration and the definition of the running-related injury used [18]. The most common injuries include patellofemoral pain, Achilles tendinopathy, iliotibial band syndrome, and plantar fasciitis, primarily affecting areas below the knee. While biomechanical factors, such as atypical lower limb alignment and altered mechanical function, have been proposed as potential contributors to RRI, the current evidence remains inconclusive, and it warrants further investigation [18]. Moreover, these non-elite athletes differ significantly from their elite counterparts in terms of physiological capacities, training volume, and performance goals. Unlike elite athletes, who often have access to professional coaching and biomechanical analyses, recreational runners are more likely to experiment with AFT independently. This autonomy could increase the risk of improper adaptation or overuse injuries, particularly if the shoes are used inappropriately or without consideration of individual biomechanics. Understanding how non-elite runners adapt to AFT, and the potential implications for injury risk and performance, is essential to developing targeted recommendations for this population.

The current evidence on how AFT influences training practices, running mechanics, and injury risk in non-elite runners is limited, making it difficult to draw definitive conclusions about its benefits and challenges for this population. Recreational runners represent a significant portion of the global running community, and understanding their response to AFT is critical, given their unique susceptibility to musculoskeletal injuries. These include acute injuries, such as muscle strains, lower limb stress, fractures, and chronic conditions like tendinopathies or joint overuse syndromes. This study aimed to address these gaps by investigating the experiences of non-elite runners using AFT, defined in this study as commercially available running shoes incorporating carbon fiber plates and innovative midsole technologies designed to enhance energy return and improve running economy. The focus of this study was on their training practices, perceived benefits, and reported challenges. Insights into how recreational runners adapt to AFT can help identify strategies to mitigate injury risks and promote safe, sustained engagement in running. By analyzing real-world data from a diverse cohort of recreational runners, this research sought to provide valuable insights into the integration of AFT into everyday training routines, offering practical recommendations for optimizing its use while minimizing potential risks.

## 2. Materials and Methods

### 2.1. Study Design

This prospective, cross-sectional, observational study was conducted following the STROBE guidelines [19]. Before completing the questionnaire, all participants provided informed consent outlining the study protocol. Data collection was carried out over six months, from 1 January to 30 June 2024. The study protocol received approval from the Institutional Ethics Review Committee of the Università degli Studi di Milano (Protocol No. 52/20, Attachment 4, 14 May 2020), adhering to the Declaration of Helsinki and all relevant ethical regulations for research involving human participants.

### 2.2. Participants

Non-elite runners training for or competing in distances ranging from 5 km to marathon length were recruited in person through running clubs located in Lombardy, Italy, and online via email and WhatsApp. The recruitment materials explicitly invited runners of varying experience levels and training practices to ensure diversity within the sample. The inclusion criteria required participants to be members of the Italian Athletic Federation (FIDAL) and to have used AFT in training or competition for distances between 5 km and a marathon. For the purposes of this study, AFT was defined as commercially available running shoes incorporating carbon fiber plates and innovative midsole technologies designed to enhance energy return and improve running economy. Participants were asked to confirm their use of AFT via a structured questionnaire, which also collected details about specific shoe models, contexts of use (e.g., training or competition), and any alternation with other footwear technologies. To minimize potential selection bias, no exclusion criteria were applied, and recruitment efforts targeted runners across different age groups and genders. Demographic and training characteristics were monitored during recruitment to avoid the overrepresentation of highly experienced or competitive runners. While the study relied on a convenience sample, this approach was designed to reflect the diversity of the recreational running population.

### 2.3. Survey

Participants were explicitly instructed to reflect on their most recent training practices and experiences to minimize recall bias. The survey was developed on Google Forms based on a review of the existing literature and validated through expert consultation. Three experts in sports biomechanics and running injuries reviewed the survey to ensure content validity. A pilot test was conducted with 10 recreational runners to assess the clarity and comprehensiveness of the questions, as well as the time required for completion. Feedback from the pilot test was used to refine the survey. To ensure reliability, selected questions were rephrased and repeated within the survey, and consistency in the responses was analyzed. Participants were provided with detailed instructions emphasizing the importance of accurate and truthful self-reporting. Anomalies or inconsistencies in responses were flagged and verified with participants when possible. Participants completed a survey comprising three main sections, designed to be completed in 10–15 min. The full survey contents, including the questions used to assess training practices, footwear usage, and perceived outcomes, are provided in Appendix A.

### 2.4. Statistical Analysis

Descriptive statistics were calculated for all outcome measures and reported as means ± standard deviations (SDs). The normality of data distribution for demographic variables (age, height, body mass, and BMI), running activity (competition type, years of practice, weekly sessions, and training volume), and AFT use (years of use and weekly sessions) was assessed using graphical methods and the D’Agostino–Pearson test. Since all variables showed normal distributions, parametric tests were applied. Continuous variables such as weekly training volume and weekly sessions were categorized into predefined ranges for visualization and comparison purposes. Weekly training volume was grouped into categories such as “0–30 km”, “30–60 km”, and “≥60 km”, while weekly sessions were categorized as “1–2 sessions”, “3–4 sessions”, and “>4 sessions”. The correlation between AFT use and running activity metrics was analyzed using Pearson’s correlation coefficient. The coefficient of determination (R^2^) calculated from Pearson’s correlation coefficient represents the proportion of variance in the dependent variable thatis explained by the independent variable in the model. Effect sizes were calculated to determine the proportion of variance explained by each correlation. To ensure the appropriateness of the linear models, a residual analysis was performed. Residuals were found to be randomly distributed around zero, confirming the validity of the regression models and indicating no systematic patterns. Correlation strength was categorized as negligible (0.0–0.3), low positive or negative (0.3–0.5), moderate positive or negative (0.5–0.7), high positive or negative (0.7–0.9), or very high positive or negative (0.9–1.0). Correlations were considered statistically significant when the correlation coefficient (r) exceeded 0.3 and *p*-values were below 0.05 [20]. Injury patterns and distribution data were summarized using descriptive statistics, including frequencies and percentages to illustrate the distribution of injuries based on the number of sessions using AFT shoes and the duration of AFT shoe use. Confidence intervals (95% CIs) were calculated for all reported percentages to provide an estimate of the precision of the observed proportions. Additionally, odds ratios (ORs) were calculated to assess the relative risk of injuries between groups, using one group as the reference category. The association between running experience and changes in running technique was analyzed using a chi-square test to evaluate the relationship between categorical variables. All statistical analyses were conducted using Prism GraphPad Prism software, version 11, for Windows (GraphPad Software, Version 11, San Diego, CA, USA).

## 3. Results

### 3.1. Demographic Characteristics

The survey was distributed to 84 non-elite runners who were members of local running clubs in Lombardy (Italy). The participants were recruited via in-person announcements during club meetings and through digital channels, including email and WhatsApp groups. Of the 84 runners invited, 61 participants (47 men, 77%; age: 38 ± 11 years; height: 1.76 ± 0.07 m; body mass: 66 ± 7 kg; BMI 21.4 ± 1.7 kg/m²; and 14 women, 23%; age: 38 ± 12 years; height: 1.63 ± 0.05 m; body mass: 53 ± 5 kg; BMI 19.2 ± 1.3 kg/m²) completed the survey, resulting in a response rate of 72%. All participants reported having used AFT during training or competition, making their responses directly relevant to the study objectives. Demographic data, including age, height, body mass, and calculated BMI, were self-reported by the participants.

### 3.2. Running Activity

**Figure 1:**
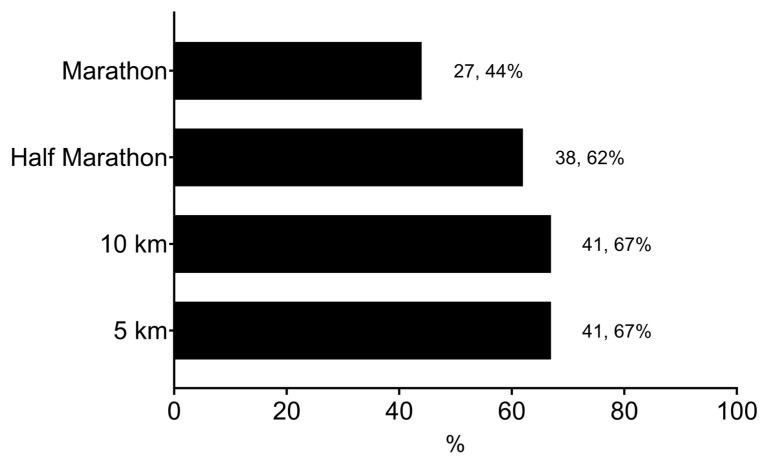
The proportion of runners reporting specific types of competition.

The participants reported competing in various distances, including 5 km (67%), 10 km (67%), half-marathon distance (62%), and marathon distance (44%). These distances represented the events for which participants were currently training at the time of completing the survey. Their average running history was 8 ± 3 years, reflecting their cumulative experience in running. Weekly training data, including the number of sessions (7 ± 3 sessions/week) and training volume (68 ± 20 km), were based on participants’ activities during the six months before the survey. Figure 1 illustrates the proportion of runners competing at each distance, including marathons, half marathons, 10 km races, and 5 km races. The percentages shown exceed 100% because many participants reported competing in multiple event types.

**Figure 2:**
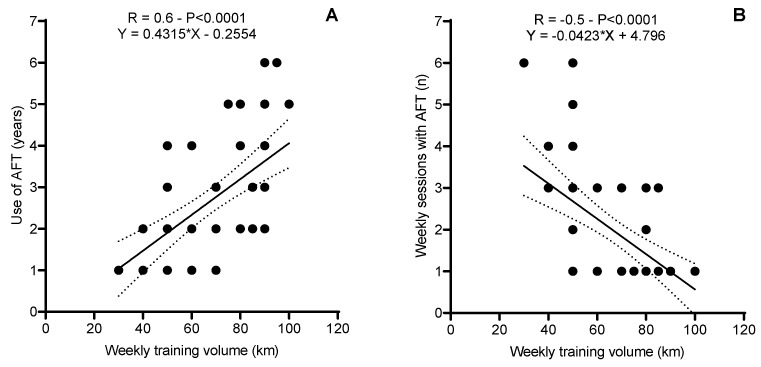
Correlation between weekly training volume and the use of advanced footwear technology (AFT) and weekly training volume (**A**) and the number of weekly training sessions (**B**). The weekly training volume was reported as continuous data and categorized into ranges (e.g., “0–30 km”, “30–60 km”, and “ ≥60 km”) for visualization purposes. Similarly, weekly training sessions were grouped into categories (e.g., “1–2 sessions” and “3–4 sessions”). The dashed lines represent the 95% confidence intervals for the regression lines.

Significant correlations were identified between weekly training volume and AFT use. A moderate positive correlation (R = 0.6, *p* < 0.0001) was found between training volume and years of AFT use, with an r^2^ of 0.4. Similarly, a moderate negative correlation (R = −0.5, *p* < 0.0001) was observed between training volume and the number of weekly training sessions using AFT, with an r^2^ of 0.3. These findings are shown in Figure 2.

Additionally, a moderate positive correlation (R = 0.6, *p* < 0.001) was observed between years of running experience and the duration of AFT use. A weak but statistically significant positive correlation (R = 0.4, *p* = 0.02) was found between running experience and the number of weekly sessions using AFT (R = 0.4, *p* = 0.02). To further support these correlations, a residual analysis was conducted. Residuals were found to be randomly distributed around zero, indicating that the linear regression models are appropriate and that no systematic patterns were observed.

Changes in running technique were analyzed by grouping participants based on their running experience (<5 years, 5–10 years, and >10 years) and categorizing reported changes into four main types: increased forefoot support, higher calf muscle activation, altered torso position, and increased instability. A chi-square test revealed no statistically significant association between running experience and reported changes in running technique (χ² = 2.31, *p* = 0.622).

### 3.3. Use of AFT in Running Activities

**Figure 3:**
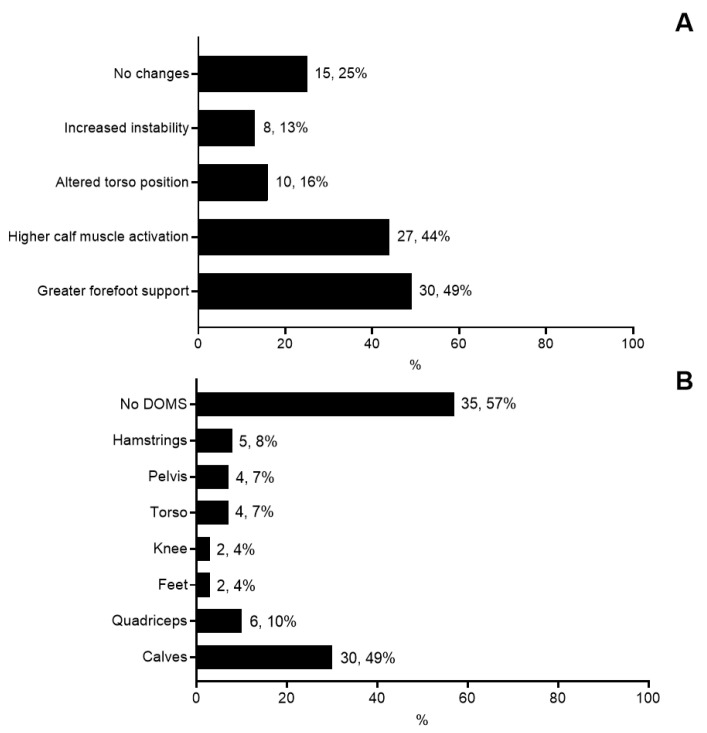
Self-reported changes in running mechanics using AFTs (**A**) and location of delayed-onset muscle soreness (DOMS) (**B**).

The average duration of AFT use was 3 ± 2 years, with 5 ± 3 weekly training sessions involving AFT. Participants reported using various shoe types, including cushioned shoes (80%), stability shoes (52%), racing flats (49%), and minimalist shoes (8%). The perceived effects of AFT use included greater forefoot support (74%), increased calf muscle activation (66%), altered torso position (56%), and heightened running instability (34%). The chi-square test showed that the distribution of self-reported changes in running mechanics was statistically significant (χ² = 37.84, *p* < 0.001). Regarding delayed-onset muscle soreness (DOMS), 49% of participants reported soreness in the posterior shank muscle strains, while fewer runners experienced DOMS in the quadriceps (10%), hamstrings (8%), pelvis (7%), or torso (7%). Overall, 57% of participants reported no DOMS after using AFT. The chi-square test showed that the distribution of soreness locations was statistically significant (χ² = 84.09, *p* < 0.001). These results are summarized in Figure 3.

### 3.4. Injury Patterns and Distribution

Eight of the sixty-one runners (13.1%) reported sustaining an injury with a diagnosis by a medical doctor after using AFT shoes. Table 1 illustrates the distribution of injuries based on the number of sessions using AFT shoes and the types of injuries sustained. Injuries were more frequent among those who used AFT shoes for up to six sessions per week, accounting for 62.5% of the cases (95% CI: 45.8–79.2%), compared to 25.8% (95% CI: 9.0–42.6%) for those using AFT shoes up to three sessions per week. The odds of sustaining an injury were approximately three times higher for runners in the “up to six sessions per week” group compared to the “up to three sessions per week” group (OR = 3.0). The injury rate in the “1 session per week” group was 100% (95% CI: 0–100%), although the small sample size (*n* = 1) limits meaningful statistical comparisons. The most common injuries included posterior shank muscle strains, calcaneal fractures, and trochanteric inflammation, with specific patterns varying by frequency of use. The “up to six sessions per week” group reported injuries such as one posterior shank muscle strain, one calcaneal fracture, one abductor tear, one trochanteric inflammation, and one case of non-specific low back pain, while the “up to three sessions per week” group experienced one pelvic stress fracture and one quadriceps tear, as noted.

Table 2 summarizes the distribution of injuries based on the duration of AFT use. The injury rate was 9.1% (95% CI: 1.1–29.2%) for runners who had used AFT shoes for less than a year, 10.0% (95% CI: 2.1–26.5%) for those using AFT shoes for 1 to 3 years, and 33.3% (95% CI: 7.5–70.1%) for those with more than 3 years of use. Compared to the reference group (1 to 3 years), the odds of sustaining an injury were lower for runners using AFT shoes for less than a year (OR = 0.5) and higher for those with more than 3 years of use (OR = 4.7). The most reported injuries included posterior shank muscle strains, pelvic stress fractures, and non-specific low back pain, observed across all groups. Specific patterns of injury varied by the duration of use, with quadriceps tears and non-specific low back pain, reported more frequently in the “1 to 3 years” group, while hip stress fractures and calcaneal fractures were more prevalent in the “more than 3 years” group.

## 4. Discussion

The use of AFT has gained popularity among non-elite runners, yet most existing research focuses on elite athletes. This leaves a significant gap in understanding how AFT influences training practices, running mechanics, and injury risks among recreational runners, who differ considerably from elite athletes in physiological capacities, training volume, and access to professional guidance. Addressing this gap, our study aimed to investigate the experiences of non-elite runners using AFT, focusing on their training practices, perceived benefits, and reported challenges. The results provide valuable insights into the perceived benefits and challenges associated with AFT while also raising questions about its long-term implications.

Our findings indicate that amateur runners who use AFT participate in a variety of competition types, ranging from 5 km races to marathons. This reflects the diverse nature of their running practices and suggests that AFT shoes are adopted across different competitive contexts to meet varying performance demands. This versatility highlights the widespread appeal of AFT among non-elite runners and reinforces the importance of understanding how these shoes impact different running scenarios. The positive correlation between weekly training volume and years of AFT use (R = 0.6, *p* < 0.0001) suggests that more experienced runners adopt AFT more consistently over time. However, the corresponding R^2^ = 0.36 indicates that only 35% of the variation in training volume can be explained by the duration of AFT use, suggesting that other factors—such as training intensity, running goals, or shoe preferences—likely contribute to this relationship. While statistically significant, the moderate explanatory power of this model highlights the importance of interpreting these findings cautiously in a clinical or practical context [21,22]. Similar trends have been reported by Bermon [9] and Hoogkamer et al. [11], who highlighted that experienced runners tend to adopt advanced footwear technologies due to their perceived benefits in energy return and reduced fatigue. However, unlike elite athletes, recreational runners often exhibit greater variability in biomechanical adaptations, as their training loads and physiological characteristics are more diverse [14]. Conversely, the negative correlation between training volume and weekly sessions using AFT (R = −0.5, *p* < 0.0001) reflects a strategic approach to footwear use. Runners appear to reserve AFT for specific sessions, likely to maximize its benefits during key workouts while minimizing potential overuse or dependence. However, the corresponding R^2^ = 0.25 indicates that only 25% of the variance in weekly AFT sessions can be explained by training volume, suggesting that other factors, such as training goals, runner preferences, or recovery strategies, may contribute to this relationship. While statistically significant, the relatively low explanatory power highlights that this finding, although meaningful, may have limited clinical significance in isolation and warrants further investigation. This finding supports recommendations by Nigg et al. [21] regarding the importance of balancing footwear technology with individual biomechanics and training goals. Additionally, the selective usage pattern observed in this study suggests that runners prioritize AFT for sessions where performance or recovery is critical, which is consistent with the findings of Malisoux et al. [15]. The high prevalence of cushioned (80%) and stability shoes (52%) suggests a focus on comfort and injury prevention, while the lower adoption of minimalist shoes (8%) underscores the technical and physical demands required for their safe use. From a practical standpoint, this behavior highlights the potential benefits of limiting AFT use to targeted sessions to optimize performance and reduce the mechanical load on specific muscle groups, such as the calves. Such an approach may also mitigate the risk of overuse injuries associated with the prolonged or inappropriate use of advanced footwear technologies. These observations underline the importance of providing recreational runners with personalized guidance to ensure that the benefits of AFT are maximized without compromising safety. Moreover, the selective use of racing flats (49%) for competitive events or speed training illustrates how runners integrate multiple footwear types into their training regimens, potentially mitigating injury risks while benefiting from performance-oriented designs. These findings emphasize the need for personalized guidance when integrating AFT into training routines, particularly for recreational runners with varying levels of experience and biomechanics.

Additionally, a moderate positive correlation (R = 0.6, *p* < 0.001) was observed between years of running experience and the duration of AFT use, indicating that more experienced runners are likely to incorporate AFT shoes into their routines over longer periods. However, the corresponding coefficient of determination (R^2^ = 0.36) shows that only 36% of the variability in AFT duration can be explained by running experience, suggesting that other unmeasured factors, such as individual biomechanics or training goals, likely contribute to this relationship. Similarly, a weak but statistically significant correlation (R = 0.4, *p* = 0.02) was observed between running experience and the number of weekly AFT sessions. In this case, the R^2^ value of 0.16 indicates that only 16% of the variance is accounted for, further underscoring the limited explanatory power of this relationship. These findings suggest that, while running experience may play a role in shaping how runners integrate AFT into their training routines, additional factors should be considered to fully understand this behavior. However, the chi-square test revealed no statistically significant association between running experience and changes in running technique (χ² = 2.31, *p* = 0.622). Despite this, descriptive trends showed that novice runners (<5 years) more frequently reported challenges such as increased instability, while experienced runners (>10 years) were more likely to report increased forefoot support and higher calf muscle activation. Intermediate runners (5–10 years) often reported altered torso positions. These observations suggest that running experience influences the biomechanical adaptations required to transition to AFT, even if these trends were not statistically significant.

Self-reported mechanical changes, including increased forefoot support (74%) and calf muscle activation (66%), highlight AFT’s impact on running mechanics. These adaptations likely stem from the longitudinal stiffness and teeter–totter effect of AFT, which shift the center of pressure forward during ground contact [22]. While these changes may improve running economy, they also appear to increase mechanical load on specific muscle groups, as evidenced by the high prevalence of DOMS in the calves (49%). The findings indicate that the biomechanical shifts associated with AFT may necessitate adaptation periods to mitigate potential discomfort or injuries. However, this observation is descriptive and does not imply causation, underscoring the need for further research to explore these potential effects [23]. The reported instability (34%) and changes in torso position (56%) among a subset of runners further underscore the variability in individual responses to AFT. These findings aligned with studies suggesting that the benefits of AFT, such as improved running efficiency, are influenced by factors including running speed, technique, and individual biomechanics. This variability highlights the importance of personalized guidance when integrating AFT into training routines.

The self-reported injury rate (16%) and diagnosed injuries (13.1%) observed in this study are consistent with general estimates of running injury prevalence [22]. However, the injuries reported, including muscle strains, bursitis, and fractures, suggest potential biomechanical contributors, such as altered load distribution and increased stiffness. While previous studies have identified parameters such as ground reaction forces and joint loading rates as risk factors for running injuries [23,24], the magnitude of these changes specific to AFT remains underexplored. The recovery period of 3 ± 2 months for diagnosed injuries underscores their severity, necessitating further investigation into the potential links between AFT and injury risk. Factors such as improper adaptation, overuse, and inadequate training may contribute to these outcomes.

This study has several limitations. First, the relatively small sample size and reliance on self-reported data limit the generalizability of the findings. Second, the study exclusively included participants who reported using AFT, precluding comparisons with non-users and limiting insights into the potential differences between AFT users and non-users in terms of training practices, injury prevalence, and biomechanical adaptations. Third, the exclusion of undiagnosed injuries may have underestimated the true prevalence of injury. Fourth, the underrepresentation of women in the sample limits the ability to draw insights into gender-specific responses to AFT despite known differences in anthropometry, biomechanics, and injury risk between men and women. Fifth, while correlations between variables were statistically significant, the explanatory power of these relationships, as indicated by r^2^ values, was moderate (36% for years of AFT use and 25% for the number of weekly sessions using AFT). This indicates that only a limited portion of the variance is explained by the measure variables, suggesting that other unmeasured factors likely play a role. Importantly, correlation analysis does not infer causality, and statistically significant findings do not necessarily translate into strong clinical implications. Finally, the absence of detailed information on pre-existing conditions and training intensity precludes a comprehensive assessment of confounding factors. Furthermore, several additional confounding variables may have influenced the results and should be considered in future studies. For instance, differences in training surface types (road or track) could affect biomechanical adaptations and injury risks. Runners training on softer surfaces may experience different biomechanical loads compared to those on harder surfaces, potentially influencing their response to AFT. Similarly, recent advancements in running shoe technology, such as the introduction of “super spikes,” have raised interest in their potential influence on performance, particularly in specific contexts like curve sprints [25]. The reliance on self-reported data also introduces the possibility of recall bias, particularly regarding injury history, training volume, and footwear usage patterns. Such bias may have affected the accuracy of the reported correlations and injury prevalence. Furthermore, the cross-sectional nature of this study limits our ability to establish causality between AFT use and the observed outcomes.

This study is among the first to document injury patterns associated with AFT, emphasizing the need for a deeper knowledge of this topic to quantify injury risks and identify mitigation strategies. Therefore, future research should aim to include larger, more diverse samples to enhance the generalizability of the findings. Longitudinal studies are needed to assess the long-term impact of AFT on performance, biomechanical adaptations, and injury risk. These studies should focus on understanding how cumulative exposure to AFT affects non-elite runners over multiple training cycles and explore adaptation differences across subgroups, such as gender, age, and training experience. Randomized controlled trials comparing AFT users and non-users will be critical to establish causality and identify effective training and adaptation protocols. Incorporating advanced biomechanical analyses, such as motion capture and pressure mapping, could provide deeper insights into how AFT influences running mechanics and injury patterns. Finally, examining the role of variables such as training surface types and intensity will help clarify the conditions under which AFT offers the most benefits. Addressing these gaps will enable the development of evidence-based guidelines to optimize the safe and effective use of AFT among recreational runners.

## 5. Conclusions

This study is among the first to investigate training practices and reported outcomes associated with AFT shoes, offering valuable insights into their potential impact on non-elite runners. The findings revealed moderate correlations between AFT use and training variables, with years of AFT use explaining 36% of the variance in weekly training volume and the number of weekly AFT sessions explaining 25%. These results highlight the strategic integration of AFT into training regimens but must be interpreted cautiously. The reliance on self-reported survey data introduces the possibility of recall bias, and the relatively small sample size, combined with the absence of a comparison group, limits the generalizability of the findings. Based on the results, we propose that non-elite runners gradually incorporate AFT shoes into their training routines to allow for adaptation to the biomechanical demands these shoes impose on the foot and lower extremities. A progressive approach may involve starting with running technique exercises, brief and gradual sprints, moving to short-distance drills, and eventually competition in long-distance running. We recommend a transition period of 4 to 8 weeks, depending on individual experience and training volume, during which runners initially integrate AFT into 1–2 weekly sessions at low intensity. Progression should be gradual, with increased duration and intensity as the body adapts. To minimize the risk of overuse injuries, runners are encouraged to monitor key indicators such as muscle soreness (particularly in the calves), discomfort, or changes in running mechanics and to adjust or suspend AFT use if symptoms persist. Incorporating strength and flexibility exercises targeting the lower extremities, especially the calf muscles and Achilles tendon, may further support adaptation and reduce injury risk. This recommendation remains exploratory, aiming to promote safe use while emphasizing the need for further research to establish evidence-based guidelines.

## Figures and Tables

**Table 1:** Distribution of injuries based on the number of sessions with AFT shoes.

*N* of Sessions with AFT Shoes	Injuries	Percentage (95% CI)	Type of Injury
**Up to 6 sessions/week**	5 out of 29	62.5% (45.8–79.2%)	1 calf tear, 1 calcaneal fracture, 1 abductor tear 1 trochanteric inflammation, 1 non-specific low back pain
**Up to 3 sessions/week**	2 out of 31	25.8% (9.0–42.6%)	1 pelvic stress fracture, 1 quadriceps tear
**1 session/week**	1 out of 1	100% (0–100%) ^a^	1 calf tear

Legend. ^a^ The injury rate in the “1 session per week” group was 100% (95% CI: 0–100%), although the small sample size (*n* = 1) limits meaningful statistical comparisons.

**Table 2:** Distribution of injuries based on the duration of AFT shoe use.

Time of Use of AFT Shoes	Injuries	Percentage (95% CI)	Type of Injury
**Less than a year**	2 out of 22	9.1% (1.1–29.2%)	1 calf tear, 1 throcanteritis
**From 1 to 3 years**	3 out of 30	10.0% (2.1–26.5%)	1 quadriceps tear, 1 abductor tear, 1 back pain
**More than 3 years**	3 out of 9	33.3% (7.5–70.1%)	1 hip stress fracture, 1 calf tear, 1 calcaneal fracture

## Data Availability

The data that support the findings of this study are available from the corresponding author upon reasonable request.

## Appendix A

The detailed structure of the survey, including questions related to demographic characteristics, training practices, footwear usage, and self-reported outcomes, is provided in Appendix A for further reference.

**Survey Structure**

The survey used in this study was structured as follows:

**1. Demographic Characteristics**

Participants were asked to self-report their demographic information, including:

- **Age**: Exact age in years at the time of survey completion.
- **Sex**: Selected gender from predefined options (men, women, or prefer not to respond).
- **Height and Body Mass**: Reported as exact measurements in meters and kilograms, respectively.
- **Body Mass Index (BMI)**: Calculated using the standard formula based on self-reported data.

**2. General Running Activity**

Participants provided details about their current running practices over the six months before completing the survey to ensure consistency and capture recent habits.

- **Running History**: Number of years of consistent training or competition (continuous variable).
- **Weekly Training Sessions**: Average number of running sessions per week during the past six months (continuous variable).
- **Distances**: Events participants were currently training for, categorized as 5 km, 10 km, half-marathon, or marathon.
- **Weekly Training Volume**: Total distance covered during a typical training week, reported as kilometers.

**3. Use of AFT in Running Activities**

Participants provided self-reported data regarding their use of Advanced Footwear Technology (AFT) shoes:

- **Duration of AFT Use**: Number of years participants had been using AFT shoes in training or competition.
- **Weekly Training Sessions with AFT**: Frequency of weekly sessions conducted using AFT shoes.
- **Use of Other Shoe Types**: Participants specified other types of shoes used during training, including:
  ○ Cushioned shoes
  ○ Stability shoes
  ○ Racing flats
  ○ Minimalist shoes
- **Perceived Reduction in Fatigue**: Participants indicated whether they experienced reduced fatigue when using AFT shoes (Yes/No).
- **Self-Reported Changes in Running Mechanics**: Participants reported biomechanical adaptations attributed to AFT shoes, including:
  ○ Increased forefoot support
  ○ Higher calf muscle activation
  ○ Altered torso position
  ○ Increased instability
- **Location of Delayed Onset Muscle Soreness (DOMS)**: Participants identified affected areas, including:
  ○ Foot
  ○ Calf
  ○ Knee
  ○ Back
  ○ Pelvis
  ○ Hip
  ○ Quadriceps
  ○ Hamstrings
- **Location of Running Injuries**: Participants reported injuries sustained while using AFT shoes and specified the affected areas.
- **Recovery Time from Injuries**: Recovery period reported in months for each injury.
- **Medical Diagnosis Details**: Participants elaborated on the type and severity of injuries and any medical interventions received (free-text field).
